# Research on Athlete Behavior Recognition Technology in Sports Teaching Video Based on Deep Neural Network

**DOI:** 10.1155/2022/7260894

**Published:** 2022-01-05

**Authors:** XianPin Zhao

**Affiliations:** Wenzhou Polytechnic, Wenzhou Zhejiang, 325003, China

## Abstract

In recent years, due to the simple design idea and good recognition effect, deep learning method has attracted more and more researchers' attention in computer vision tasks. Aiming at the problem of athlete behavior recognition in mass sports teaching video, this paper takes depth video as the research object and cuts the frame sequence as the input of depth neural network model, inspired by the successful application of depth neural network based on two-dimensional convolution in image detection and recognition. A depth neural network based on three-dimensional convolution is constructed to automatically learn the temporal and spatial characteristics of athletes' behavior. The training results on UTKinect-Action3D and MSR-Action3D public datasets show that the algorithm can correctly detect athletes' behaviors and actions and show stronger recognition ability to the algorithm compared with the images without clipping frames, which effectively improves the recognition effect of physical education teaching videos.

## 1. Introduction

With the development of society, more and more sports teaching videos have entered people's daily life. The analysis of PE teaching video can more effectively improve the teaching effect. Object detection and human pose estimation for sports video are the basis of sports video analysis and understanding. The existing target detection and human pose estimation technologies have achieved good performance in the general scene detection task based on pictures [1], but there are few algorithms and data for target detection in sports video scenes. For a new data field, accurate athlete detection and pose estimation are important links in sports video analysis. The existing human target detection and pose estimation algorithms have achieved good performance in the general human body detection task, but they will detect athletes and spectators at the same time in the physical education teaching video, so they cannot further distinguish athletes' targets, which will interfere with the subsequent video analysis. At the same time, the sports video data with human body annotation is scarce [2], and the cost of obtaining a model suitable for the field of sports video is high. Athletes are a special case of general human body detection task. If the general human body detection model can be used to detect and estimate athletes in sports video, it can undoubtedly save a lot of cost. In order to reduce the labelling cost and training cost, the detection and pose estimation of athletes in sports teaching video are realized.

Among the traditional methods, the two-dimensional behavior recognition technology based on RGB video has been widely studied [3]. The early method is to extract local spatiotemporal descriptors from the input video and encode these descriptors into word bags with visual vocabulary for classification. At the same time, the local feature method has become an effective way in motion recognition, because these local features do not need algorithms to detect the human body and are not sensitive to factors such as background, illumination, and noise. The calculation of these local features can usually be divided into two parts. The calculation of these local features can usually be divided into two parts: detector and descriptor. Detector is to find the significant and informative region of action understanding and descriptor, and descriptor is to describe the visual model of the extracted region. Laptev proposed spatiotemporal points of interest by extending Harris angle to spatiotemporal dimension [4], and Kviatkovsky et al. proposed a covariance descriptor to realize online action recognition [5]. Local descriptors for video representation include histogram of oriented gradient (HOG) features and histograms of optical flow (HOF) features [6]. The two-dimensional convolution neural network used for RGB static pictures cannot meet the requirements of extracting time features. Another method is to apply the three-dimensional convolution neural network, but the three-dimensional neural network has a great disadvantage that there are too many parameters to be optimized, so it needs a large-scale dataset for training [7].

Inspired by the successful application of depth neural network based on two-dimensional convolution in image detection and recognition, this paper takes physical education video as the research object and constructs a depth neural network based on three-dimensional convolution to automatically learn the temporal and spatial characteristics of human behavior, which is used for human behavior recognition. Combining convolution neural networks and recurrent neural networks, convolution neural networks are used to extract spatial features, and then cyclic neural networks are used for time series modeling. This method can avoid the demand for optical flow and better meet the real-time requirements. Taking the deep learning method as the key technology, VGG16 convolutional neural network is used to extract the highly abstract features of video frames, and an athlete behavior recognition algorithm in sports teaching video is designed and implemented.

## 2. Related Work

At the beginning of this century, some foreign universities began to pay more attention to the research of human behavior recognition. Today, a lot of research has been carried out in the field of human behavior recognition [8], and more and more valuable research results have emerged. The recognition methods have made rapid progress and changed with each passing day. With the rise of deep learning, the research on human behavior recognition using deep neural network has begun to be more and more in depth. Compared with foreign countries, China started late in this area, and there is still a certain gap to catch up. In recent years, it has begun to strengthen research. Davis and Taylor [9] first used contour to describe the global feature of human body, which is a two-dimensional feature. Motion energy image (MEI) is used to indicate the position where motion has occurred, motion history image (MHI) is used to indicate the spatial position and time sequence of motion, and then recognized by Mahalanobis distance classifier, these two kinds of feature images are obtained by background frame difference method. The optical flow method based on pixel level can capture and locate the moving object. When the background frame difference method cannot extract the foreground object well, the optical flow method can be used for recognition and location. It is easy for the optical flow method to introduce the motion noise of noninterested objects. Literature [10] only calculates the optical flow at specific positions (such as the approximate center point of the object), which reduces the influence of noise to a certain extent.

Blank et al. [11] first obtained 3D spatiotemporal volume (STV) from silhouette information in video or picture sequence, then derived local spatiotemporal interest points and their directional features through Poisson equation, and obtained a 3D global feature by weighting these local points. In contrast to the global feature, the local feature adopts the bottom-up research thinking, and only relatively independent image blocks in the object region are extracted. Since the region of interest (ROI) [12] does not have to be a complete object with practical significance in the real world, accurate object region positioning and tracking are not required. The usual approach is to calculate some spatiotemporal interest points in the video or picture sequence, then expand and fuse them into image blocks one by one, and finally combine all image blocks to generate total features. Because it does not depend on the accurate positioning and tracking of the bottom layer, the sensitivity of local features to noise, angle change, and occlusion is not high. However, due to the need for a large number of stable interest points related to action categories, various complex preprocessing processes cannot be avoided. At the same time, the motion of the camera will also affect the calculation and detection of interest points. In the traditional human behavior recognition algorithm, it is necessary to artificially control the complex process of feature extraction and data reconstruction. By using the deep neural network, the picture can be directly used as the input of the network, and the recognition effect can be better than that of the traditional method [13]. Deep neural network does not completely break away from the traditional methods and start a new stove but integrates the complex steps of the traditional methods into the network and simplifies the whole process. All connections and parameters in the network reflect some ideas of the traditional methods. Compared with the traditional shallow neural network, deep neural network, as its name implies, has more network layers in structure and a large number of parameters, and its internal connection mode will change greatly with the different types of networks. The multilayer structure of the network enables it to learn higher-level and more abstract expressions [14], so that the features extracted from the input data will have higher discrimination.

Human posture estimation is also called human joint point regression, which detects the position of each joint point of the human body and then connects it to the human skeleton. Human posture estimation is not only the basis of behavior analysis and motion recognition but also an important part of video analysis [15]. If the human joint points are returned to 3D for expansion, RGB images are input, and 3D human key points are output, it is 3D human pose estimation. This is a very valuable research direction. The traditional human posture estimation algorithm divides the human body into multiple parts according to joints, hand, head, leg, and body and then performs feature matching based on the manually extracted features of each part to detect the limbs in the figure. Taking the body as the trunk, the detected limb parts are spliced according to the Gaussian model of human posture distribution to obtain a complete human posture [16]. This method needs to manually design human features, and the feature engineering steps are complex. When the scene is complex, it is difficult to cover all the situations, which has great limitations and cannot be effectively popularized. With the development of deep learning, human pose estimation algorithm based on deep learning has become the mainstream. The human posture estimation algorithm based on deep learning regards human posture detection as a key point regression problem, trains through a large number of data with joint point category and position annotation, and finally obtains a model that can predict the position and category of human joint points [17].

The existing deep learning models mainly include convolutional neural networks (CNNs), recurrent neural networks (RNNs), restricted Boltzmann machine (RBM), and deep belief network (DBN) [18]. These methods give full play to the self-learning ability of neural network and let the network adapt to the characteristics of data. The method of manually selecting features is avoided. A large number of studies show that these models can achieve better results than the original methods in handwritten numeral recognition, target detection and recognition, speech recognition, and other scenes.

### 2.1. Athlete Behavior Recognition Model Based on Deep Neural Networks

#### 2.1.1. Athlete Behavior Tracking

There are strong rules in shooting and editing techniques in sports video. The characteristics of different shots and the differences between different shots are obvious. In the far shot, the competition field accounts for the largest proportion, and the athletes are also concentrated on the competition field. Therefore, the competition field area is detected first. After the athletes are detected, the more accurate athletes can be obtained by removing the field color by using the consistent color characteristics of the sports field. In the middle lens, the field color still accounts for a certain proportion in the image, so if the far lens scene has been processed, the main field color is stored to remove the nonathlete area of the image belonging to the competition field.

**Figure 1:**
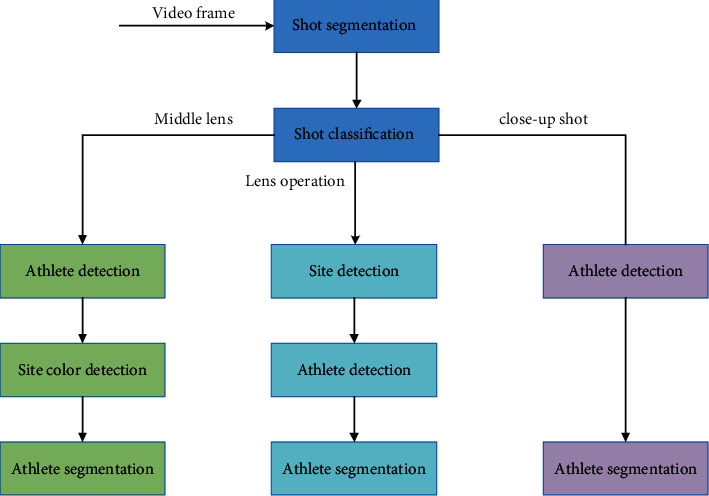
Monitoring and segmentation of athletes in sports teaching video.

Athlete detection is a classifier that combines the characteristics of athletes and background in various types of shots in sports video and uses middle-level feature blocks to train and distinguish athletes. The middle-level characteristics of neural network often contain rich information, which plays a connecting role in the whole neural network. In this paper, CamShift algorithm is used to track athletes, and its core algorithm is mean shift algorithm. Mean shift algorithm is a nonparametric probability density estimation method. Its algorithm block diagram is shown in Figure 1. It finds the peak points in the probability distribution through the iterative process and makes each point “drift” to the local maximum of the density function. CamShift algorithm is an extension of mean shift algorithm. It realizes the tracking of moving objects in continuous video frames by adaptively adjusting the size and position of search window. It has the characteristics of good robustness, fast speed, and less computation. Using kernel function estimation method, when the sampling is sufficient, it can gradually converge to any density function; that is, it can estimate the density of data subject to any distribution. In this way, the convergence of mean shift algorithm can be guaranteed, and the mean shift vector can be used for iteration. It can better track the irregular motion of nonrigid objects and has good antinoise ability.

An important step in CamShift algorithm is to convert the original video image into a probability distribution map by using the backprojection of the color histogram of the target object. Each pixel value in the map represents the probability that the pixel belongs to the target object. Because HSV color space separates color (H) from saturation (S) and brightness (V), using the H channel of HSV color space to establish histogram can reduce the sensitivity to illumination changes. The traditional method of calculating histogram is as follows:

(1) $$\begin{array}{c} q_{u}=\sum_{x\in R}\sum_{y\in R}c\delta \left(x,y\right)-u^{2}, \end{array}$$

where {*q*_*u*_}*u*=1,2,…, *m* is the histogram; *R* is the target area where histogram needs to be calculated; the function *C*: *R*_2_ ⟶ {1, 2,…, *m*} is used to convert the pixel value with coordinates of (*x*, *y*) into the histogram level value; and *δ* is the Kronecker symbol:

(2) $$\begin{array}{c} \delta \left[i-j\right]=\left\{\begin{array}{c} 0,\text{other}\\ 1,i=j \end{array}\right.. \end{array}$$

In order to make the obtained probability distribution map within the range of [0255], the histogram needs to be scaled:

(3) $$\begin{array}{c} p_{u}=255\cdot \min\left(q_{u}q_{\max}^{-1},1\right), \end{array}$$

where *u* = 1, 2, ..., *m*; *q*_max_ = {max(*q*_*u*_)}*u* = 1, 2, ..., *m*. Let the value of the pixel at (*x*, *y*) in the video image be *I* (*x*, *y*) and *p* be the probability distribution map corresponding to the image, then, *P*(*x*, *y*)=*P*_*f*[*I*(*x*, *y*)]_, where {*P*_*u*_}, *u* = 1, 2, ..., *m* is the histogram; function *f* [*I* (*x*, *y*)] finds the corresponding level value in the histogram according to the value of *l*(*x*, *y*).

CamShift is a semiautomatic tracking algorithm, which needs to explicitly specify the initial size and position of the search window. In order to realize automatic tracking, this paper takes the detected athlete area as the initial search window. In the iterative process, the *i* centroid (*x*_*c*_, *y*_*c*_) of window *w* is

(4) $$\begin{array}{c} x_{c}==\sum_{x\in w}\sum_{y\in w}xP\left(x,y\right){\left[\sum_{x\in w}\sum_{y\in w}P\left(x,y\right)\right]}^{-1},\\ y_{c}=\sum_{x\in w}\sum_{y\in w}yP\left(x,y\right){\left[\sum_{x\in w}\sum_{y\in w}P\left(x,y\right)\right]}^{-1}. \end{array}$$

Each iteration resizes the window to 2^−3^[∑_*x*∈*w*_∑_*y*∈*w*_*P*(*x*, *y*)]^1/2^.

Because the traditional histogram only uses the information of H channel, it is easy to fail to track when the hue distribution of the target object is similar to that of the background.

In order to make CamShift's tracking algorithm more robust, this paper uses the weighted histogram method to improve the algorithm. In the athlete's area, the pixel farther away from the center of the area has lower reliability, and the pixel is more likely to be covered by other objects or belong to the background. Therefore, it is reasonable to assign a relatively low weight to the pixel far away from the center of the area when calculating the histogram. The weighted histogram is calculated as follows:

(5) $$\begin{array}{c} q_{u}=\sum_{x\in w}\sum_{y\in w}k\left(r\right)\left[c\delta \left(x,y\right)-u^{2}\right], \end{array}$$

where *K*(*r*) is a monotonically decreasing function, (*x*_*c*_, *y*_*c*_) is the regional center, and $r=\sqrt{{\left(x-x_{c}\right)}^{2}+{\left(y-y_{c}\right)}^{2}}$. In order to ensure the real-time tracking, this paper sets the number of iterations of each mean shift as 15 times, and the convergence standard is to drift one pixel.

#### 2.1.2. Behavior Feature Extraction Based on VGG16 Model

The feature extraction layer of VGG16 convolution neural network model is used to process the frame picture. The input of the model is the cropped frame picture, which needs to be normalized to 224 × 224; this paper takes the output of the feature extraction part, which is a 4096-dimensional vector. Each picture in the frame sequence needs feature extraction and finally forms a feature sequence.

**Figure 2:**
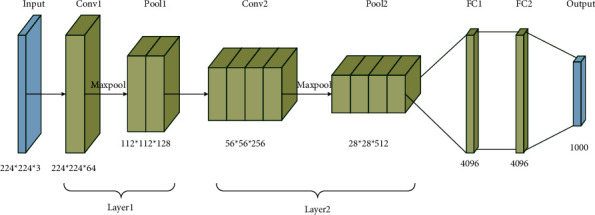
VGG16 network model structure diagram.

VGG networks are divided into several classes according to the number of layers. The structures of all classes are very consistent, and almost all convolution layers use 3 × 3 size convolution kernel (only one class uses 1× the convolution kernel with the size of 1 being regarded as a linear mapping of space), and the pooling layer also adopts 2 × 2 size aggregation. Using a smaller convolution kernel can reduce a considerable part of the parameters under the action of weight sharing. In shallow convolution neural network, less parameters often lead to insufficient generalization ability of the model, resulting in underfitting. At this time, the network parameters can be effectively increased and the expression ability of the model can be improved by superimposing convolution layer. In addition, it also creates conditions for more nonlinear mapping and enhances the fitting ability of the network. Common VGG networks usually have 16–19 convolution layers. There are obvious groups between these convolution layers. At the end of each group, there is a pool layer, and the output of this group is downsampled. VGG16 network model is adopted in this paper, with a total of 16 convolution layers. Its structure is shown in Figure 2.

In Figure 2, there are 5 groups of convolution layers, and the size of convolution kernel is 3 × 3. The first group has two convolution layers, each layer has 64 convolution cores to extract features from multiple angles and dimensions, the second group has two layers, 128 convolution cores per layer, the third group has three layers, 256 convolution cores per layer, and the fourth and fifth groups also have three layers, 512 convolution cores per layer. Each group of convolutional layers is followed by a 2 × 2 pooling layer. At the end of the network is a 3-layer fully connected layer. The entire network has a total of 138 million parameters to be trained.

In terms of training, VGG16 will first perform necessary preinitialization of some layers, including include_top: whether to include the top fully connected layer; weights: None stands for random initialization of “ImageNet,” which stands for pretrained weights loaded on ImageNet; input_tensor: Optional, Keras tensor is used as the input of the model (that is, the tensor output by layers.Input()); input_shape: Optional, input size tuple, valid value when only include_top = False; pooling: Optional. When include_top is False, this parameter specifies the pooling method for feature extraction. In addition, the depth of the network and the small size of the convolution kernel have the effect of implicit regularization, so it only needs a few iterations to start convergence. This paper uses the VGG16 model fully trained on ImageNet, because its input scale is fixed at 224 × 224, so the pictures in the frame sequence need to be reduced to the corresponding size before they can be sent to the network. The length width ratio of the cropped frame picture is about 219 × 214, so there will not be too much distortion after zooming. In addition, the network itself has certain scale invariance, so it can be put into the network for feature extraction.

In terms of calculation, as mentioned above, the convolution layer mainly convolutes or performs correlation operations on the pictures, the pooling layer mainly subsamples each feature image obtained from the previous layer to reduce its size and retain useful information while reducing the amount of data, and the full connection layer integrates local features to achieve higher abstraction or data classification or prediction (the last layer is the full connection layer). Like most deep neural networks, the activation units of all convolution layers in the network adopt the modified linear unit (ReLU) activation function. The activation function of artificial neuron generally adopts nonlinear monotone function which is continuous and differentiable everywhere, and the value range of the function is a fixed interval. The requirement of nonlinearity is that the linear function can only play the role of linear amplification of the input, and the linearity will greatly reduce the performance of the network, and it is easy to degrade the multilevel network function into a single-level network function, which is a fatal defect. The training algorithm can calculate the gradient and adjust the parameters by using the gradient. Monotonic performance can effectively improve the efficiency of network training. The bounded value range that can be given by the actual demand can make the gradient decline more stable and prevent the network from entering the saturation state: if the input is generally small, the value range can be adjusted to have a larger gain. If the input is generally too large, adjust the value range to get a smaller gain.

The activation effect of ReLU function is similar to that of biological neurons, and its mathematical expression is shown as follows:

(6) $$\begin{array}{c} f\left(x\right)=\left\{\begin{array}{cc} 0 & x<0,\\ x & x\geq 0. \end{array}\right.. \end{array}$$

**Figure 3:**
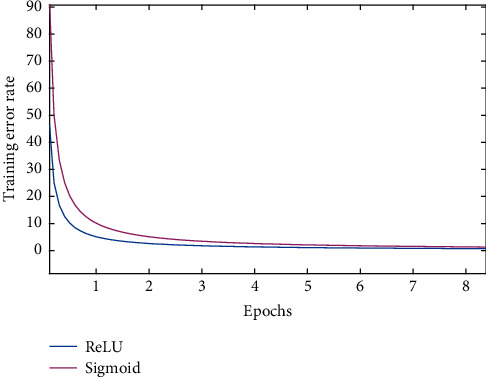
Comparison of convergence effect.

As shown in Figure 3, the blue line and red line, respectively, represent the convergence effect of ReLU activation function and sigmoid activation function. It can be seen from the figure that the convergence speed of training model with ReLU activation function is significantly faster than that with sigmoid activation function, which greatly shortens the learning cycle.

Deep learning is actually a feature driven method. As a typical method of deep learning, the network model composed of convolutional neural network is no exception. Usually, the output of the feature extraction layer of the network can be taken out separately as a feature description of the data, and these feature descriptions can be used to classify or predict the data in other ways. In VGG16, after layer by layer calculation, the input image gradually transitions from local feature extraction to global feature construction. The output of each layer can be taken out separately as a feature description. The last three layers of the network are fully connected to the network layer, except the last layer (used to directly process the features extracted by the previous layer to classify or predict the data) that can well describe the global features of the image. Take the output of the first fully connected layer, whose output form is 4096-dimensional vector, and the extracted feature description will be used as the input of the next layer network.

#### 2.1.3. Training Method of Athlete Classifier in Sports Teaching Video

In sports video, the characteristic of athletes and background is that all pictures contain background and athletes, and there are basically no frame images without athletes. In addition, in addition to close-up shots, athletes account for a small proportion of the frame images in other shots, and most of the frame images are background blocks. Moreover, the background in the sports scene is the competition venue, and the competition venue structure of the same type of sports competition is very similar. In this case, it is not feasible to mine middle-level feature blocks by unsupervised iterative clustering detection. In the initial clustering, due to the large number of background blocks and unified structure, most of the classes with less than or equal to three image blocks in the discarded class are foreground blocks. The cohesive clustering algorithm makes decisions according to the local pattern without considering the global structure of the data. Therefore, cohesive clustering is very suitable for identifying smaller classes. When using unsupervised iterative clustering detection method, the first thing to do is to separate the background from the athlete block. Because the sample collection stage can only be separated manually, the workload is too large.

From another point of view, these characteristics of athletes in sports video can simplify the definition of middle-level feature blocks. In the close-up shot, the athlete only has a bust, and there are not many types of athletes. There are not many kinds of blocks that contain different parts of athletes. In the middle shot and far shot, the proportion of athletes in the image is small; especially in the far shot of football, tennis, and badminton, most athletes can use a small block for complete segmentation. The number of blocks with large difference between athletes in the image segmentation of middle lens and far lens is not very much. Based on this feature, the middle-level feature block that can distinguish athletes and nonathletes is defined as the block with the most athlete contour. All qualified blocks are used as the positive samples of the training athlete classifier, and other blocks are used as the negative samples of the training athlete classifier. The following will explain how to collect positive samples and negative samples to train the athlete classifier in the sports video.

**Figure 4:**
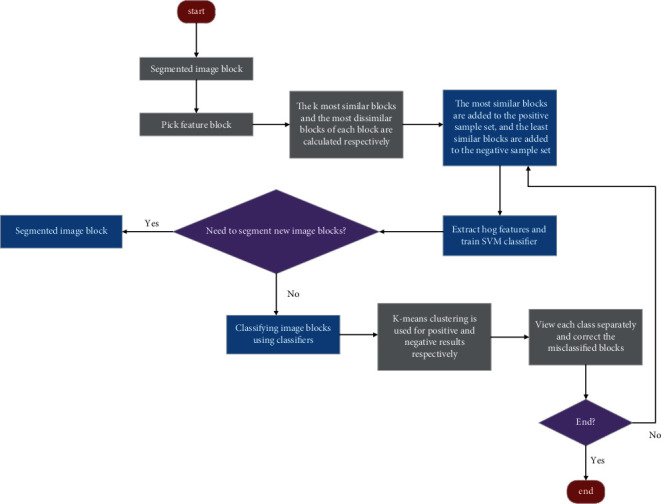
Process of training athlete classifier.

The steps of collecting positive and negative samples of the classifier for training athletes are shown in Figure 4. It should be noted that, in order to calculate the most similar blocks and the *K*-means clustering results in the classification results more accurately, the pictures of each segmented block are from the same video. The image is segmented with a fixed window and step size to obtain the image block set. The image blocks with the largest athlete contour and different parts are selected as seed blocks, and the *k* most similar blocks and *k* least similar blocks of each seed block are calculated, respectively. Taking all the blocks most similar to the seed block as the positive sample set and the least similar block as the negative sample set, the hog features of all positive and negative sample blocks are extracted, and the SVM classifier is used to train the athlete's classifier, which is the initial athlete classifier. In the later iteration, the classifier is used to classify and detect all blocks, and then *K*-means is used to cluster the pictures with positive and negative results. Manually check whether the classification corresponding to each clustering result is wrong. If it is wrong, add the wrong class to the correct sample, so as to expand the positive and negative sample set. At the beginning of the next iteration, reextract the features of the sample set and train a new classifier, which optimizes the classifier. Iteratively detect, cluster, and update the optimized classifier until the classifier correctly classifies a certain number of new video picture sets.

#### 2.1.4. Athlete Behavior Recognition Model and Overall Recognition Process Based on Deep Neural Network

**Figure 5:**
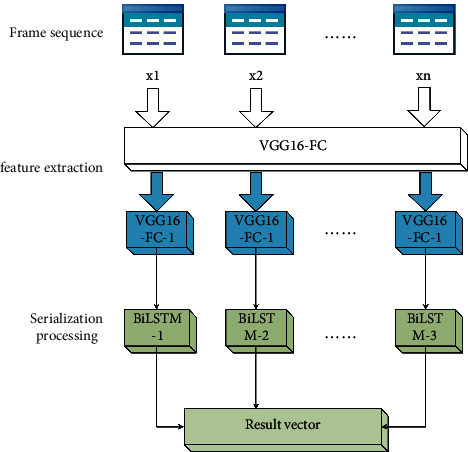
Model diagram of deadlock detection method for hierarchical scheduling system based on time constraints.

Combining the feature extraction of spatial dimension and the information integration of time dimension, we combine to form a VGG16-BiLSTM athlete behavior recognition model, as shown in Figure 5.

**Figure 6:**
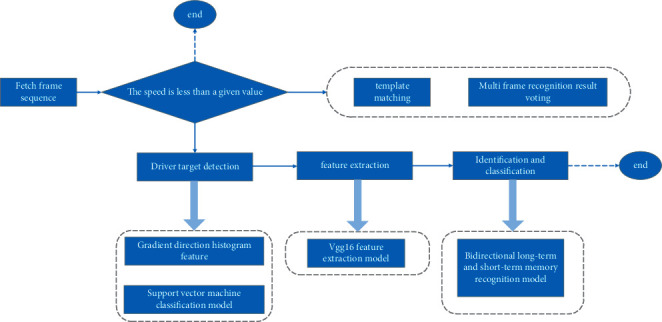
Flowchart of the identification process in the final athlete behavior detection system.

The model takes the cropped frame sequence as the input and the output vector of the last time of the cyclic neural network as the result. The first part of the model is the feature extraction layer of VGG16 deep convolution neural network, which adopts the weight parameters fully trained on ImageNet and takes the output of its first full connection layer as the extracted image features. The second part is the bidirectional long-term and short-term memory model with more advantages in integrating time dimension information. Its weight parameters are trained with the feature sequence extracted from the real shooting data of physical education teaching video used by the subject as the training data. It takes the feature sequence obtained in the first part as the input and outputs the recognition results of the whole model. Integrating all the above research contents, Figure 6 shows the overall identification process in the final athlete behavior detection system.

## 3. Experiment and Analysis

### 3.1. Experimental Setup and Pretreatment

Two public datasets, UTKinect-Action3D and MSR-Action3D, are used to evaluate the proposed method. They are the behavior video data of physical education teaching video captured by Kinect. There are 10 behaviors in UTKinect-Action3D, namely, walk, sit down, stand up, pick up, carry, throw, push, pull, wave hands, and clap hands. There are 10 PE teaching videos, each behavior in each teaching video twice, and a total of 199 effective videos. For the convenience of calculation, all 200 videos are used. There are 20 behaviors in MSR-Action3D dataset, which are shot by 10 subjects, in which each athlete completes each behavior 2-3 times.

According to the experimental settings in the basic project research of the PE teaching video dataset, 20 behaviors are divided into three behavior subsets, AS1, AS2, and AS3 (as shown in Table 1). Each behavior subset contains 8 different behaviors.

In order to reduce the impact on different experimental results, each video is simply preprocessed before the experiment:

1. Background removal: in depth video, the depth information of the background is consistent, while the depth information of the foreground is changed. The background information can be removed according to this feature. Gaussian model is a common method of background removal in video image detection. This method is suitable for dynamic video image feature detection, because the foreground and background are separated in the model. The benchmark for separating foreground and background is to judge the change rate of pixels. The learning with slow change will be regarded as the background, and the learning with fast change will be regarded as the foreground.
2. Determination of bounding box: for each video, the bounding box that can and can only frame the athlete's behavior is obtained according to each frame, and the maximum bounding box of all frames is taken as the bounding box of this video (see Figure 2).
3. Normalization: use interpolation technology to normalize all videos processed in the previous step to a unified size, where the number of normalized video frames is equal to the middle value of all video frames. At the same time, the min max method is used to normalize the depth information values of all videos to the range of [0, 1]. Finally, all samples are turned horizontally to form new samples, so as to multiply the training samples in the dataset. After preprocessing, the behavior video sizes of UTKinect-Action3D and MSR-Action3D are 28 ∗ 32 ∗ 32 and 38 ∗ 32 ∗ 32, respectively, including the number of frames, frame width, and frame height in the video from front to back. The experiment depth neural network model part is compiled by torch platform, and the data preprocessing part is completed by Matlab platform.

### 3.2. Performance of Athlete Behavior Recognition Algorithm Based on Deep Neural Network

**Figure 7:**
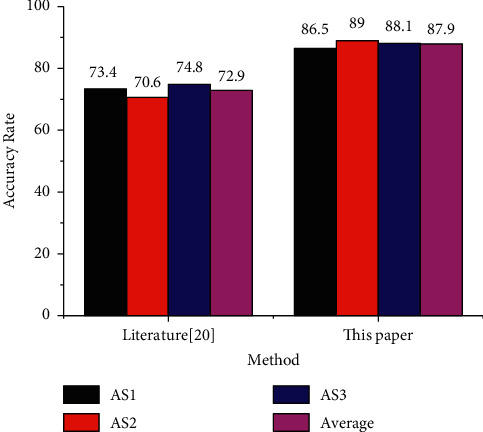
Comparison with the benchmark project in MSR-Action3D dataset (%).

Firstly, the effectiveness of the research method is verified on the MSR-Action3D dataset. According to the experimental setting of [19], the research method is compared with the benchmark project research of the dataset [19] and several main methods based on artificial feature extraction in recent years.  Figure 7 shows the method and the accuracy of behavior recognition on three different behavior subsets. From the recognition results, it can be seen that the athlete behavior recognition method based on 3D convolution depth neural network can effectively recognize human behavior, and the recognition accuracy and average accuracy of each behavior subset are better than the benchmark research of the dataset.

**Figure 8:**
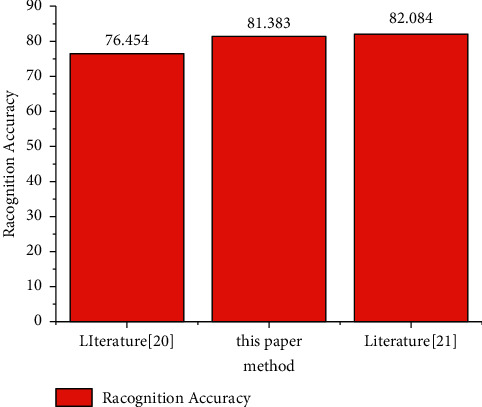
Performance evaluation compared with [20, 21] (%).

This is mainly because the 3D word bag model is used to extract the features in the behavior video. This feature can extract the representative 3D word bag information in the physical education teaching video but ignores the spatial and temporal information in the video. When doing video evaluation experiments, we need to select representative video sequences according to certain conditions. Temporal information (Ti, temporal perceptual information, also known as temporal complexity) and spatial information (Si, spatial perceptual information, also known as spatial complexity) can be used to measure the characteristics of video. Where Si is high, there are more spatial details of video frames (high spatial complexity). Where Ti is high, the video frame content moves violently (with high time complexity). The athlete behavior recognition method based on 3D convolution depth neural network adopts 3D convolution operation for the video, which effectively maintains the spatial and temporal features. Therefore, better performance is obtained.  Table 2 shows the comparison between the research method and the current method with the best recognition efficiency [20]. The experiment adopts the setting of the method in [20]. The results show that the proposed method can maintain consistent recognition performance with the methods in [20], which fully shows the effectiveness of this method (Figure 8).

In the UTKinect-Action3D dataset, the research method is compared with the benchmark research project on the dataset. Literature [21] used leave-one-out cross validation method (LOO-CV). For the convenience of the experiment, the study uses leave one subject out cross validation (LOSO-CV); that is, only one subject's all behavior videos are used as the test set at a time, while the data of other subjects are used as the training set, so as to train a deep neural network model for each subject. Obviously, the experimental conditions are more stringent than those in [20].

It can be seen from Table 2 that the average accuracy rate of behavior recognition of each subject is 80.125%, which can basically correctly identify the behavior of most subjects. However, the recognition accuracy rate on physical education teaching videos 5, 6, 7, and 10 is relatively low, which may be due to the large deviation of perspective of athletes in these sports teaching videos when shooting action behavior because UTKinect-Action3D is a multiperspective dataset. However, there is still a certain gap between the research method and the 90.92% recognition rate in [21], with a difference of nearly 8 percentage points. It may be because it makes full use of the skeleton information in the depth video and uses hidden Markov model (HMM) to establish the temporal model of skeleton information. Its disadvantage is that the behavior recognition framework is too complex, and the system performance is affected by skeleton information extraction and HOL3D feature extraction, feature LDA projection, behavior word clustering, HMM model training, and other links, and literature [21] can see that skeleton extraction is a complex process, and the accuracy of the extracted skeleton information depends on the shooting of depth video. At the same time, the experimental conditions of the research method are more stringent than those in [20], the experimental data are relatively small, and the training of the model is insufficient, which are also the reasons for the poor recognition effect. Nevertheless, compared with the methods of feature extraction such as [21], the method based on deep learning has better generalization performance, and the research method does not need complex artificial feature extraction links. It only needs simple processing of the original video, and the deep neural network model can automatically extract features and complete the recognition and classification process. The method is simple and involves fewer links.

#### 3.2.1. Behavior Recognition Parameter Experiment

If the extracted frame image sequence is directly sent to the network model for training, recognition, and classification, the recognition effect will not be ideal because of too many interference factors. Direct training can not model complex video, and the video modeling effect with long time is not good. Although the recognition subject in the video is an athlete, other moving objects appear in the picture due to the angle and complex actual scene during video shooting, and their dynamic changes will interfere with behavior recognition. Therefore, in the process of preprocessing the data, the support vector machine is used to detect the gradient direction histogram characteristics of each region in the frame picture to locate the athlete's region and then cut off the picture information outside the region of interest. After the model is trained, the multiscale detection method is used to detect the target in a picture. The multiscale method uses the image pyramid and sliding window, which involves two important parameters: the downsampling scaling factor scale: the divisor of the width and height of the image divided by each downsampling.

Sliding step means that how many pixel units the sliding window slides each time it slides horizontally or vertically. The variation range of detection scale directly affects the detection effect and consumption time. Setting these two parameters reasonably is a key to optimize the recognition effect and recognition time. In the sequence data, the “sliding window” is used to intercept sequence fragments, thereby reshaping the original data into samples of a specified length for model modeling. On the premise of achieving the best detection effect, we should reduce the scale range as much as possible to reduce the detection time.

**Figure 9:**
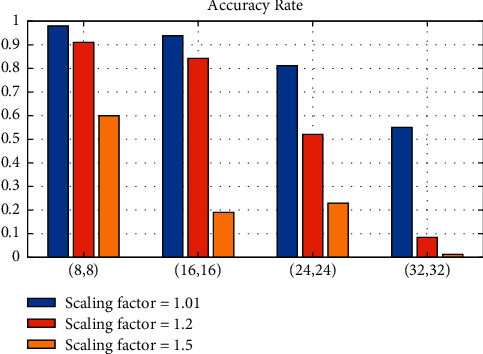
Accuracy rate of multiscale detection (%).

100 frame pictures from different videos are selected as the test data. Taking the ability to correctly detect the athlete's area and further obtain the athlete's area as the standard for successful recognition, two variables are controlled for detection, respectively. The detection results are shown in Figure 9, in which the ordinate represents the scale factor, and the abscissa represents the sliding step of the sliding window in the form of (horizontal sliding step, vertical sliding step).

**Figure 10:**
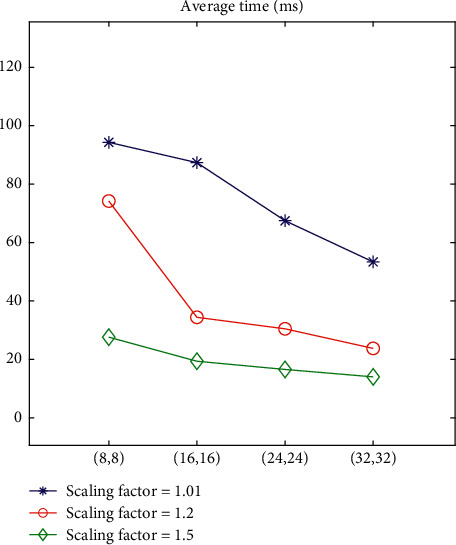
Average time of multiscale detection (ms).

It can be seen from the table that the average detection time is inversely correlated with the scaling factor and sliding step size. Considering the data in Figures 9 and 10, it is finally decided to select the scaling factor of 1.2 and the sliding step size of (16, 16).

**Figure 11:**
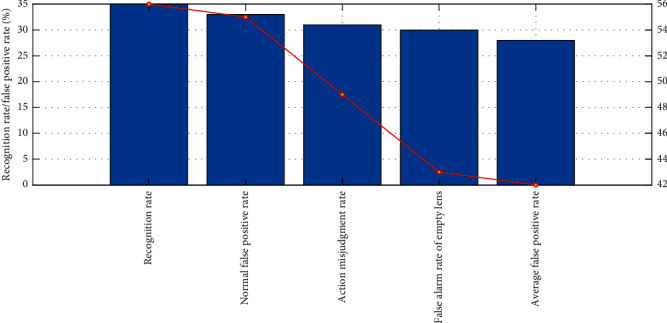
Comparison of recognition effect before and after cutting.

The experimental data are 250 training videos and 60 test videos. All videos take 30 frames at equal intervals and then use VGG16 network model to extract features and train and test through two-way long-term and short-term memory network model. Under the control of other variables, the frame sequences before and after clipping are used, respectively, for experiments; the contrast effect before and after clipping is shown in Figure 11. After cutting, the overall recognition effect has been significantly improved, the recognition rate has increased from 42% to 27%, the single category false alarm rate has decreased, and the average false alarm rate has decreased by nearly half, as low as 9%.

## 4. Conclusion

This research is based on a deep neural network and uses the CamShift algorithm to track athletes. It collects positive and negative samples of the training athlete's classifier and extracts the HOG features of all positive and negative sample blocks. CamShift can effectively solve the problem of target deformation and occlusion. Resource requirements are not high, time complexity is low, and good tracking results can be achieved in a simple background. However, when the background is more complicated, or there are many pixels that are similar to the target color, it will cause the tracking to fail. Therefore, the SVM classifier is used to train the athlete's classifier and uses the algorithm of cutting frame pictures and VGG16 model to recognize athletes' behavior. After some interference information in the picture is cut out in the data preprocessing of the system, in order to further strengthen the attention of the network model to the actions of athletes' key parts, the mechanism of adding attention in the cyclic neural network can be used to further improve the recognition effect. Although some progress has been made in improving model reusability and reducing labelling and training costs, there are still many aspects that can be improved, such as improving the speed of sports video target detection and human posture estimation. The adjacent frames in the video have very high similarity. This paper uses this to fuse the multiframe results, which improves the detection accuracy, but also brings a lot of redundant detection. In the later stage, we can consider accelerating the detection according to the redundant information between frames.

## Figures and Tables

**Table 1:** The action subsets AS1, AS2, and AS3 in MSR-Action3D dataset.

AS1	AS2	AS3
Horizontal arm wave	High arm wave	High throw
Hammer	Hand catch	Forward kick
Forward punch	Draw x	Side kick
High throw	Draw tick	Jogging
Hand clap	Draw circle	Tennis swing
Bend	Two-hand wave	Tennis serve
Tennis serve	Forward kick	Golf swing
Pickup & throw	Side boxing	Pickup & throw

**Table 2:** The action recognition accuracy of each subject in UTKinect-Action3D.

Subjects	Subject 1	Subject 2	Subject 3	Subject 4	Subject 5	Subject 6	Subject 7	Subject 8
Recognition rate%	87	85	89	81	76	72	78	73

## Data Availability

The data used to support the findings of this study are available from the corresponding author upon request.

## References

[1] Jalal A., Akhtar I., Kim K. (2020). Human posture estimation and sustainable events classification via pseudo-2D stick model and K-ary tree hashing. *Sustainability*.

[2] Fasel B., Spörri J., Schütz P., Lorenzetti S., Aminian K. (2017). An inertial sensor-based method for estimating the athlete’s relative joint center positions and center of mass kinematics in alpine ski racing. *Frontiers in Physiology*.

[3] Byeon Y.-H., Kim D., Lee J., Kwak K.-C. (2021). Ensemble three-stream RGB-S deep neural network for human behavior recognition under intelligent home service robot environments. *IEEE Access*.

[4] Laptev I. (2005). On space-time interest points. *International Journal of Computer Vision*.

[5] Kviatkovsky I., Adam A., Rivlin E. (2012). Color invariants for person reidentification. *IEEE Transactions on Pattern Analysis and Machine Intelligence*.

[6] Colque R. V. H. M., Caetano C. (2016). Histograms of optical flow orientation and magnitude and entropy to detect anomalous events in videos. *IEEE Transactions on Circuits and Systems for Video Technology*.

[7] Wood A., Mack R., Turner M. (2020). Developing self-determined motivation and performance with an elite athlete: integrating motivational interviewing with rational emotive behavior therapy. *Journal of Rational-Emotive and Cognitive-Behavior Therapy*.

[8] Bo T.-B., Zhang X.-Y., Kohl K. D., Wen J., Tian S.-J., Wang D.-H. (2020). Coprophagy prevention alters microbiome, metabolism, neurochemistry, and cognitive behavior in a small mammal. *The ISME Journal*.

[9] Davis J. W., Taylor S. R. (2002). Analysis and recognition of walking movements, object recognition supported by user interaction for service robots. *IEEE*.

[10] Aslani S., Mahdavi-Nasab H. (2013). Optical flow based moving object detection and tracking for traffic surveillance. *International Journal of Electrical, Computer, Energetic, Electronic and Communication Engineering*.

[11] Blank M., Gorelick L., Shechtman E. Actions as space-time shapes.

[12] Ashraf R., Afzal S., Rehman A. U. (2020). Region-of-Interest based transfer learning assisted framework for skin cancer detection. *IEEE Access*.

[13] Donker T., Cornelisz I., Van Klaveren C. (2019). Effectiveness of self-guided app-based virtual reality cognitive behavior therapy for acrophobia: a randomized clinical trial. *JAMA Psychiatry*.

[14] Fitzpatrick K. K., Darcy A., Vierhile M. (2017). Delivering cognitive behavior therapy to young adults with symptoms of depression and anxiety using a fully automated conversational agent (Woebot): a randomized controlled trial. *JMIR mental health*.

[15] Käll A., Jägholm S., Hesser H. (2020). Internet-based cognitive behavior therapy for loneliness: a pilot randomized controlled trial. *Behavior Therapy*.

[16] Wright J. H., Mishkind M., Eells T. D., Chan S. R. (2019). Computer-assisted cognitive-behavior therapy and mobile apps for depression and anxiety. *Current Psychiatry Reports*.

[17] Lindner P. (2021). Better, virtually: the past, present, and future of virtual reality cognitive behavior therapy. *International Journal of Cognitive Therapy*.

[18] Agras W. S. (2019). Cognitive behavior therapy for the eating disorders. *Psychiatric Clinics of North America*.

[19] Kreuze L. J., Pijnenborg G. H. M., de Jonge Y. B., Nauta M. H. (2018). Cognitive-behavior therapy for children and adolescents with anxiety disorders: a meta-analysis of secondary outcomes. *Journal of Anxiety Disorders*.

[20] Forsell E., Bendix M., Holländare F. (2017). Internet delivered cognitive behavior therapy for antenatal depression: a randomised controlled trial. *Journal of Affective Disorders*.

[21] Brotto L. A., Bergeron S., Zdaniuk B., Basson R (2020). Mindfulness and cognitive behavior therapy for provoked vestibulodynia: mediators of treatment outcome and long-term effects. *Journal of Consulting and Clinical Psychology*.

